# Evaluating Performance and Perceptions of Phase I Bachelor of Medicine and Bachelor of Surgery (MBBS) Students Toward the Objective Structured Practical Examination (OSPE) in Clinical Biochemistry: A Cross-Sectional Observational Study

**DOI:** 10.7759/cureus.109514

**Published:** 2026-05-23

**Authors:** Kapil Raghuwanshi, Vikas Rangare, Bhupendra Kumar Jain, Mahendra Gandhe

**Affiliations:** 1 Department of Biochemistry, Chhindwara Institute of Medical Sciences, Chhindwara, IND; 2 Department of Medicine, Chhindwara Institute of Medical Sciences, Chhindwara, IND; 3 Department of Respiratory Medicine, Chhindwara Institute of Medical Sciences, Chhindwara, IND

**Keywords:** assessment, biochemistry, india, mbbs students, objective structured practical examination, perception and performance of ospe, phase i mbbs students, student perception

## Abstract

Background and objective

Assessment methods in medical education should ensure objectivity, consistency, fairness, and competency-based evaluation of students. Conventional practical examinations are often limited by examiner bias, variability in questioning, and lack of standardization. The Objective Structured Practical Examination (OSPE) has emerged as a structured alternative for assessing practical and analytical skills in medical education. This study aimed to assess the feasibility and consistency of OSPE implementation and evaluate student perceptions toward OSPE in clinical biochemistry among Bachelor of Medicine and Bachelor of Surgery (MBBS) Phase I students.

Methodology

This cross-sectional observational study was conducted among 150 MBBS Phase I students in the Department of Biochemistry at Chhindwara Institute of Medical Sciences, Madhya Pradesh, India. Participants were randomly allocated into three equal groups (n=50 each) and assessed using a 10-station OSPE in clinical biochemistry. Standardized checklists and uniform station instructions were used across all groups. Student performance was recorded as mean scores, and perceptions regarding OSPE were evaluated using a pre-validated structured feedback questionnaire administered anonymously and voluntarily after the examination. Statistical analysis was performed using one-way ANOVA and chi-square tests, with p<0.05 considered statistically significant.

Results

The mean OSPE scores were comparable across all three groups, with no statistically significant difference observed (Group A: 45.32 ± 0.21; Group B: 45.33 ± 0.24; Group C: 45.36 ± 0.22; F=0.436, p=0.6474), suggesting consistency in examination conduct across batches. Student perceptions toward OSPE were favorable. Most participants reported that OSPE was well organized (90%), fair and unbiased (87%), and preferable to conventional practical examinations (90.7%). Clear station instructions were acknowledged by 93% students, while 88% reported improved confidence in practical skills.

Conclusion

OSPE was found to be a feasible, structured, and learner-accepted assessment approach in clinical biochemistry. Comparable performance across student groups suggests consistency in implementation, while students perceived the examination as organized and fair. However, the present study primarily evaluated student perceptions and operational feasibility. Further comparative and psychometric studies are required to establish the reliability, validity, and long-term educational impact of OSPE compared with conventional assessment methods.

## Introduction

Assessment is an essential component of the teaching-learning process in medical education, as it influences learning behavior, skill development, and knowledge acquisition [1]. Effective assessment strategies must ensure fairness, consistency, and comprehensive evaluation of competencies. Although traditional practical examinations are widely used, they are often affected by examiner bias, variability in questioning, and subjective marking patterns [2]. These limitations may compromise the reliability and validity of such examinations. Consequently, structured assessment tools such as the Objective Structured Practical Examination (OSPE) have been introduced for more standardized evaluation in laboratory-based disciplines.

OSPE is an adaptation of the Objective Structured Clinical Exam (OSCE) principles for laboratory-based assessment [3]. It is structured and objective, utilizing standardized checklists across multiple stations to assess specific competencies [4].

In clinical biochemistry, theoretical knowledge is integrated with laboratory skills, data interpretation, and clinical correlation, which are not fully assessed by conventional methods [5]. OSPE enables evaluation across cognitive, psychomotor, and affective domains [6]. It is considered more reliable, less examiner-dependent, and more acceptable to students than traditional assessment methods [7,8]. However, the effectiveness of any assessment strategy is also influenced by student perception [9], and evaluating both learner perception and performance outcomes provides valuable insights for curriculum design [10].

OSPE provides a systematic framework for assessing clinical reasoning, practical competence, and analytical ability in clinical biochemistry, where interpretation of laboratory data in a clinical context is essential [5,8]. Within the Competency-Based Medical Education (CBME) framework, which emphasizes outcome-based and integrated learning, the early use of OSPE for assessing laboratory skills among Bachelor of Medicine and Bachelor of Surgery (MBBS) Phase I students offers an opportunity to evaluate both conceptual understanding and its clinical application [10]. The structured and objective design of OSPE is derived from the core principles of the OSCE [11]. However, there is limited evidence regarding the early implementation of OSPE in clinical biochemistry in the newly established Central Indian medical colleges [12] . Against this background, the present study was undertaken to evaluate the effectiveness of OSPE among Phase I medical students.

In addition, evidence comparing OSPE with conventional practical examinations in terms of competency achievement and long-term learning outcomes remains limited in this setting. Therefore, the present study was undertaken to assess the feasibility and consistency of OSPE implementation and evaluate student perceptions toward OSPE in clinical biochemistry among MBBS Phase I students.

## Materials and methods

Study design and participants

This cross-sectional observational study was conducted in the Department of Biochemistry at Chhindwara Institute of Medical Sciences, Madhya Pradesh, over one month (December 2025). Ethical approval was obtained from the Institutional Human Ethics Committee of the Chhindwara Institute of Medical Sciences (approval no. CIMS/Ethics Committee/2024/11659; dated October 23, 2024). A total of 150 MBBS Phase I students from the 2025-2026 academic batch were included after obtaining written informed consent.

The sample size (n=150) was determined based on the total number of eligible Phase I students enrolled during the study period to ensure complete coverage. Participants were randomly assigned to three equal groups (n=50 each) using a computer-generated random number list based on roll numbers. The grouping was performed primarily for logistical management of the OSPE process and to assess consistency of examination conduct across different student batches. The OSPE blueprint is used for student assessment (Table 1).

**Table 1:** OSPE blueprint for phase I MBBS clinical biochemistry Total Marks=50, Allotted time=60 minutes (06 minutes for each station). OSPE: Objective Structured Practical Examination (OSPE); MBBS: Bachelor of Medicine and Bachelor of Surgery; BCG: Bromocresol Green; CBME: Competency-based medical education.

Station No.	Station type	Task	Assessment focus	CBME competency domain	Marks
1	Procedural	Serum albumin estimation by BCG method	Observation, calculation, and clinical interpretation	Skill	5
2	Procedural	Examination of abnormal urine	Performance of tests, observations, and report preparation	Skill	5
3	Interpretation	Thyroid profile	Diagnosis and conceptual understanding	Knowledge & Application	5
4	Interpretation	Rothera’s test	Interpretation, principle, and clinical use	Knowledge & Application	5
5	Calculation	Specific dynamic action (SDA)	Numerical problem-solving and significance	Application	5
6	Case-based	Scurvy	Clinical diagnosis and biochemical correlation	Knowledge & Application	5
7	Spotter	Urinometer	Identification and use	Knowledge	5
8	Spotter	Fluoride (gray-top) tube	Identification, use, and reference values	Knowledge	5
9	Spotter	Centrifuge	Identification, principle, and application	Knowledge	5
10	Good laboratory practice	Biomedical waste management	Laboratory safety and ethics	Professionalism	5

Assessment of performance

The OSPE consisted of 10 stations, each of six-minute duration and carrying five marks, with a total score of 50. These stations assessed multiple competency domains, ensuring a balanced evaluation of knowledge, application, skills, and professionalism. Scores were recorded as mean ± standard deviation (SD) for each group.

Two procedural stations assessed practical biochemical skills, including abnormal urine examination (BI 11.8, BI 11.9) and serum albumin estimation using the bromocresol green (BCG) method (BI 11.11, BI 11.12). Two interpretation stations evaluated analytical ability and clinical correlation through thyroid profile interpretation (BI 7.5, BI 7.6) and Rothera’s test (BI 11.10). One calculation-based station assessed metabolic application competencies using particular dynamic action (BI 6.2). One case-based station integrated nutritional biochemistry and clinical presentation using a scurvy case (BI 4.4, BI 4.5). Three spotter stations evaluated fundamental laboratory skills related to centrifuges (BI 11.2), fluoride blood collection tubes (BI 11.3), and urinometer identification (BI 11.1). One station on Good Laboratory Practice assessed professionalism, laboratory safety, and biomedical waste segregation (BI 11.15).

Overall, the OSPE blueprint aligned with competency-based medical education (CBME) objectives by emphasizing competency-driven, objective, and reproducible evaluation of Phase I MBBS students in clinical biochemistry. 

The OSPE was conducted uniformly for all students using identical station sequences, standardized instructions, and structured checklist-based assessment. A total of five faculty examiners from the Department of Biochemistry participated in the assessment process. The same examiner remained assigned to the same station throughout the examination to minimize inter-examiner variability. All examiners underwent orientation regarding station objectives, scoring criteria, and checklist-based marking prior to examination conduct.

Student feedback questionnaire

Following the assessment, a pre-validated structured feedback questionnaire comprising eight items was administered to evaluate student perceptions of OSPE. Participation in the feedback survey was voluntary. Students were informed that responses would remain anonymous and would not influence academic performance or examination results. Three senior faculty members performed content validation. Internal consistency was assessed using Cronbach’s alpha (0.81), indicating good reliability. The questionnaire is given in Table 2.

**Table 2:** Questionnaire for student feedback on OSPE OSPE: Objective Structured Practical Examination.

Serial number	Questionnaire item	Response options
1	Was the OSPE well-run and organized?	Yes/ No/Can’t say
2	Was the OSPE conducted in an impartial and equitable manner?	Yes/ No/Can’t say
3	Were the instructions for the OSPE clear and easy to understand?	Yes/ No/Can’t say
4	Was the time allotted to each OSPE station sufficient?	Yes/ No/Can’t say
5	Did the OSPE reduce examiner bias and ensure consistent assessment?	Yes/ No/Can’t say
6	Did the OSPE boost your confidence in practical skills?	Yes/ No/Can’t say
7	Was the OSPE less stressful and did it help you focus better on learning?	Yes/ No/Can’t say
8	Did you prefer the OSPE format over traditional examination methods?	Yes/ No/Can’t say

Statistical analysis

Categorical data were summarized as n (%), while continuous data were expressed as mean ± SD. One-way ANOVA was used to compare mean scores among groups, with p<0.05 considered statistically significant. The chi-square test was used for categorical variables. Statistical analysis was performed using Microsoft Excel (Microsoft Corporation, Redmond, USA).

## Results

Table 3 shows that students had a highly positive perception of OSPE in clinical biochemistry.

**Table 3:** Student perceptions towards OSPE in clinical biochemistry OSPE: Objective Structured Practical Examination.

Serial number	Students' perception item	Yes; N (%)	No; N (%)	Can’t say; N (%)	Non- responder; N (%)
1	The OSPE operated efficiently and was well-organized	135 (90%)	4 (2.7%)	5 (3.3%)	6 (4%)
2	The OSPE was carried out in an unbiased and fair manner	131 (87%)	6 (4%)	7 (4.7%)	6 (4%)
3	The instructions at each station were clear, succinct, and simple to follow	140 (93%)	2 (1.3%)	2 (1.3%)	6 (4%)
4	Each station had enough time to finish tasks	134 (89%)	5 (3.3%)	6 (4%)	5 (3.3%)
5	OSPE reduced examiner bias and ensure consistent assessment	129 (86%)	6 (4%)	7 (4.7%)	8 (5.3%)
6	The OSPE increased my confidence in practical skills	132 (88%)	7 (4.7%)	4 (2.7%)	7 (4.7%)
7	The OSPE was less stressful than traditional exams	130 (86.7%)	4 (2.7%)	5 (3.3%)	11 (7.3%)
8	I favored OSPE over conventional examination techniques.	136 (90.7%)	6 (4%)	6 (4%)	2 (1.3%)

Most students reported that the examination was well organized and efficiently conducted, with 135 (90%) responding positively. A total of 131 (87%) students considered the assessment fair and unbiased, while 140 (93%) reported that the instructions at each station were clear and easy to follow. Adequate time to complete tasks was reported by 134 (89%) students. Furthermore, 129 (86%) students agreed that OSPE minimized examiner bias and ensured consistency in assessment. A total of 132 (88%) students reported improved confidence in practical skills. Additionally, 130 (86.7%) perceived OSPE as less stressful than traditional examinations, and 136 (90.7%) preferred OSPE over conventional examination methods.

Table 4 shows the comparison of student performance in OSPE among the three groups.

**Table 4:** Comparison of the marks obtained in the OSPE by students from all three groups OSPE: Objective Structured Practical Examination.

OSPE performance	Group	N	Mean ± SD	Test statistic (df)	p-value
Marks obtained (out of 50)	Group A	50	45.32 ± 0.21	F = 0.436, df =147	0.6474
	Group B	50	45.33 ± 0.24		
	Group C	50	45.36 ± 0.22		

The mean scores (out of 50) were 45.32 ± 0.21 for Group A, 45.33 ± 0.24 for Group B, and 45.36 ± 0.22 for Group C. Statistical analysis showed no significant difference in performance among the groups (F=0.436, df=147; p=0.6474), indicating comparable performance across all groups.

## Discussion

This study evaluated the implementation of the OSPE in clinical biochemistry among MBBS Phase I students and assessed both student performance consistency and learner perceptions. The findings demonstrated comparable mean scores across the three randomly allocated student groups, suggesting consistency in examination conduct and scoring procedures. In addition, students reported highly favorable perceptions regarding organization, fairness, clarity of instructions, reduced stress, and confidence in practical skills.

The favorable student perceptions observed in the present study are consistent with previous reports demonstrating improved acceptability and perceived objectivity of OSPE in medical education. Townsend et al. [11] reported that structured practical assessments improve competency-driven learning by aligning assessment tasks with defined learning objectives. Similarly, Wani et al. [12] and Gujjala et al. [13] observed that the use of structured stations and standardized checklists in OSPE helps reduce variability associated with conventional practical examinations and improves consistency of assessment procedures. The perception among students that OSPE was fair, organized, and less examiner-dependent in the present study also aligns with findings reported by Diwan et al. [14] and Menezes et al. [15], who demonstrated higher student satisfaction and perceived objectivity with OSPE-based assessments across different medical disciplines.

The structured station format, checklist-based marking, and uniform task allocation used in the present study may have contributed to minimizing examiner-related variability and ensuring consistency in examination conduct across the three student groups. Furthermore, the use of competency-oriented tasks involving interpretation, procedural skills, calculations, spotters, and professionalism aligns with the principles of CBME, which emphasize observable and measurable learning outcomes.

However, the findings of the present study should be interpreted cautiously. Although students perceived the assessment as fair and objective, the study primarily evaluated operational feasibility, consistency of implementation, and learner perceptions rather than the complete psychometric effectiveness of OSPE. The absence of statistically significant differences in mean scores among the three groups supports consistency of examination administration but does not independently establish reliability or validity of the assessment method. Additional psychometric analyses, such as examiner agreement, inter-rater reliability, internal consistency, and validity testing, would be necessary to conclusively establish OSPE as a standardized and reliable assessment tool.

In addition, no control group assessed through conventional practical examination methods was included in the current study. Therefore, conclusions regarding the superiority or equivalence of OSPE compared with traditional practical examinations cannot be established. Although students expressed a preference for OSPE over conventional examinations, learner satisfaction alone is insufficient to determine competency achievement or long-term educational effectiveness. Future prospective comparative studies involving conventional assessment groups may provide stronger evidence regarding the relative effectiveness of OSPE in competency evaluation, knowledge retention, and clinical application.

The reduced stress perception reported by students in the present study may be attributed to the structured and predictable examination format, which minimizes uncertainty during assessment. Similar findings have been reported by Lakshmipathy et al. [16], who observed that repeated exposure to structured practical assessments improved student confidence and engagement in competency-based learning environments. Early integration of OSPE within undergraduate medical education may therefore support competency-oriented practical learning and enhance familiarity with structured clinical reasoning approaches.

Overall, the findings of the present study support the feasibility and learner acceptability of OSPE implementation in clinical biochemistry within a newly established medical college setting. However, larger multicenter studies incorporating comparative assessment models, psychometric reliability analysis, and long-term competency outcomes are required before definitive conclusions regarding the superiority or standardization of OSPE can be established.

Strengths 

The study utilized a structured OSPE blueprint aligned with CBME competencies across cognitive, psychomotor, and professionalism domains. Uniform station conduct, examiner orientation, and standardized checklists improved consistency of implementation. Inclusion of both objective performance scores and student feedback allowed assessment of operational feasibility and learner acceptability. The study also demonstrates the feasibility of implementing OSPE in a resource-constrained educational setting.

Limitations

The present study primarily evaluated student perceptions and consistency of OSPE implementation and did not include psychometric analyses such as inter-rater reliability, internal consistency, or validity assessment. Therefore, conclusions regarding reliability and standardization should be interpreted cautiously. The study was conducted at a single institution with a homogeneous student population, limiting generalizability.

In addition, no control group assessed through conventional practical examination methods was included; therefore, the relative effectiveness of OSPE compared with traditional practical examinations could not be determined. Long-term retention of knowledge and competency outcomes were also not evaluated.

Although students were instructed not to discuss station content, the possibility of inter-student communication regarding OSPE stations during sequential assessment sessions cannot be completely excluded. Future studies may address this limitation using multiple station sets or parallel OSPE circuits.

Implications

Faculty development will be vital in implementing change; it will alter the role of the instructor, who will not be a lecturer, but a facilitator. The gap in knowledge between theory and practice in OSPE can be bridged early on in training through emphasis on hands-on reasoning to enhance clinical preparedness. The ability to integrate interdisciplinarily also facilitates the achievement of CBME’s purpose of outcome-based competency education. The study provides a significant level of student approval and performance improvement that policymakers and educators can use to justify increased utilization of OSPE in undergraduate medical programs. To enhance metacognitive skills, clinical reasoning, and lifelong learning attributes in accordance with the CBME framework, the introduction of structured reflective practice and self-assessment elements in future implementations will be necessary. Further studies on retention and transfer of knowledge gained using OSPE should adopt multiple methods of assessment and follow students over extended periods of time.

## Conclusions

The present study demonstrates that OSPE is a feasible, structured, and learner-accepted assessment approach in undergraduate clinical biochemistry. Students perceived the examination as organized, fair, and helpful in improving confidence in practical skills, while comparable scores across student groups suggest consistency in examination implementation.

However, the present study primarily reflects operational feasibility and student perceptions rather than definitive evidence of psychometric effectiveness. Since no comparison with conventional practical examinations was performed, conclusions regarding superiority of OSPE in competency assessment should be interpreted cautiously. Further multicenter comparative studies incorporating reliability testing, validity assessment, and long-term competency outcomes are required before establishing OSPE as a fully standardized and superior alternative assessment method in undergraduate medical education.
